# From Diagnosis to Decision-Making: A Systematic Review of the Management of Reverse Hill-Sachs Lesions after Posterior Shoulder Dislocations

**DOI:** 10.3390/jcm13072085

**Published:** 2024-04-03

**Authors:** Vito Gaetano Rinaldi, Federico Coliva, Antongiulio Favero, Domenico Alesi, Silvio Caravelli, Stefano Zaffagnini, Giulio Maria Marcheggiani Muccioli

**Affiliations:** 1 II Orthopaedic and Traumatologic Clinic, IRCCS Istituto Ortopedico Rizzoli, Via Pupilli 1, 40136 Bologna, Italy; vitogaetano.rinaldi@ior.it (V.G.R.); antongiulio.favero@ior.it (A.F.); domenico.alesi@ior.it (D.A.); stefano.zaffagnini@unibo.it (S.Z.); giulio.marcheggiani2@unibo.it (G.M.M.M.); 2 Bentivoglio Orthopaedic Unit, IRCCS Istituto Ortopedico Rizzoli, Via Pupilli 1, 40136 Bologna, Italy; silvio.caravelli@ior.it; 3 Dipartimento di Scienze Biomediche e Neuromotorie DIBINEM, University of Bologna, Via San Vitale, 40125 Bologna, Italy

**Keywords:** posterior shoulder dislocation, McLaughlin, reverse Hill Sachs, allograft, shoulder instability, shoulder dislocation

## Abstract

(1) **Background**: The aim of this study is to describe all of the possible surgical procedures that intend to treat the McLaughlin lesion (or Reverse Hill-Sachs) in posterior shoulder dislocation. (2) **Methods**: Google Scholar, Pubmed, and Embase were used as databases in our research. Studies reporting the results of posterior shoulder dislocations surgically treated with procedures addressing the humeral lesion were evaluated. The studies reporting results after fracture—dislocation and multidirectional instability were excluded. (3) **Results**: A total of 16 studies were included in our review for a total of 207 shoulders with a mean age of 41.7 years that were evaluated at a mean of 62.1 months. The Modified McLaughlin procedure and the Graft procedures were the most commonly performed. No statistically significant difference was found between the two at the evaluation of the clinical score. (4) **Conclusions**: Our review highlights the importance of a correct diagnosis and an accurate surgical treatment choice based on the surgeon’s experience and on the patients’ characteristics.

## 1. Introduction

Posterior shoulder dislocations represent a relatively small fraction of all shoulder dislocations, accounting for approximately 2–4% of cases [1] and embodying a distinct clinical challenge due to their often subtle presentation and associated osseous injuries.

Among the spectrum of injuries associated with anterior shoulder dislocations, the reverse Hill-Sachs lesion remains a distinctive, yet challenging, pathology to treat. This well-documented sequela of posterior shoulder dislocation, described first by McLaughlin in 1952, is an impaction fracture at the anteromedial aspect of the humeral head, which results from its forceful contact with the posterior glenoid rim during the dislocation event [2].

The implications of reverse Hill-Sachs, also known as McLaughlin lesions, are profound. Left untreated or inadequately managed, these lesions can lead to persistent joint instability, pain, decreased range of motion, and the early onset of degenerative changes [3]. The breadth of lesion sizes, ranging from small defects occupying a small percentage of the humeral head to extensive ones encompassing a significant portion, adds another layer of complexity to clinical management.

Over the years, multiple diagnostic techniques have emerged, striving to accurately assess the lesion’s depth, size, and position, which all dictate the therapeutic approach. With many available treatments, from conservative management to surgical procedures, selecting the optimal intervention becomes paramount.

Several surgical approaches have been proposed, depending on the severity of the condition, the patient’s health status, and other factors.

One of the oldest, yet effective, methods is the subscapularis transfer into the humeral head defect, also known as the McLaughlin Procedure [2].

With the advent of arthroscopic techniques, minimally invasive options like cartilage elevation, capsulolabral repair, and plications have become increasingly popular [4,5].

In severe cases, with a humeral defect involving 30–50% of the humeral articular surface, allograft or autograft bone grafting has been proposed [6,7,8].

Furthermore, in elderly patients, partial or total shoulder replacement may be the most effective option [9,10,11].

Moreover, the consequences of a missed or inadequately treated McLaughlin lesion can be grave, including persistent pain, limited range of motion, joint instability, and a potential progression to osteoarthritis.

Furthermore, the choice of treatment, be it conservative management, surgical repair, or humeral head replacement, is often determined by the size and location of the defect, the chronicity of the lesion, and the demands of the patient [6].

Biomechanical studies have demonstrated that posterior shoulder instability is significantly influenced by both soft tissue and osseous lesions. Advanced imaging techniques, such as Magnetic Resonance (MR) arthrography and Computed Tomography (CT) scans, have been shown to be particularly effective in accurately evaluating these anatomical factors [12].

Understanding McLaughlin lesions entirely—their pathophysiology, clinical implications, and treatment modalities—is crucial for optimal patient outcomes.

Despite the extensive body of literature on this subject, there remains a paucity of consensus regarding the optimal management strategies for reverse Hill-Sachs lesions. It is still unknown what treatment provides the best clinical and radiological results and how the treatment indications vary according to the lesion characteristics and the clinical history of the patient. This systematic review aims to collate and critically analyze existing studies on posterior shoulder dislocation, with a particular focus on understanding the natural history and the outcomes of various treatment modalities. Through this endeavor, we hope to shed light on evidence-based best practices, identify gaps in our current understanding, and provide a structured framework for clinical decision-making.

## 2. Materials and Methods

### 2.1. Literature Search

The literature search was performed on 1 December 2023 by three reviewers (V.G.R., F.C., A.F.). Pubmed, Google Scholar, and Embase were used as the databases for our research. The string used for our Pubmed and Embase research was “(((reverse Hill-Sachs) OR (McLaughlin)) AND (treatment)) AND (shoulder dislocation)” while for Google Scholar, we used the advanced search tool with “posterior shoulder dislocation treatment” as an exact phrase, including at least one of these words “Reverse Hill-Sachs OR McLaughlin” and without the words “fracture dislocation”. 

**Figure 1:**
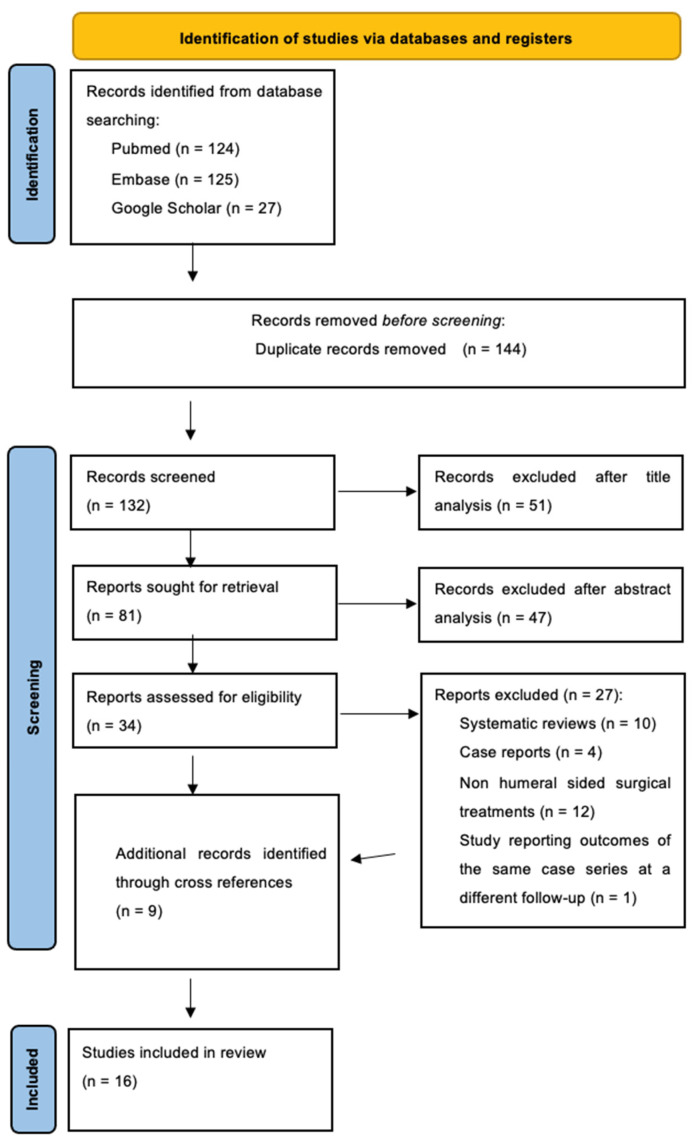
Prisma Flowchart.

All relevant studies between 2003 and 2023 were identified in accordance with the Preferred Reporting Items for Systematic Review and Meta-Analysis (PRISMA) guidelines (Figure 1 and Prisma Checklist in Supplementary Materials).

We included in our analysis all the papers that were screened as inherent to our review topic by at least two of the three reviewers. All three reviewers were actively engaged in the article selection process for this systematic review, initially screening based on article titles and subsequently conducting thorough abstract analysis. This method ensured a rigorous and objective selection process, minimizing the likelihood of omissions or errors in selecting studies pertinent to the review. 

All studies between 2003 and 2023 were identified in accordance with the Preferred Reporting Items for Systematic Review and Meta-Analysis (PRISMA) [13] guidelines (Figure 1 and Prisma Checklist in Supplementary Materials). The first analysis was conducted on the article titles. The studies were then checked through a careful analysis of the abstract. The authors also evaluated the bibliographies of the included articles, especially the included systematic reviews, to search for further studies that were added later to our review if they met all the inclusion and exclusion criteria. 

All the selected articles adhered to the Population, Intervention, Comparison, and Outcomes (PICOS) criteria for systematic reviews [14]. 

### 2.2. Research Protocol

Our research protocol was registered on PROSPERO (CRD42023490511) after the literature search was completed. Once the approval of the protocol was obtained, the data extraction and analysis began. 

### 2.3. Eligibility Criteria

The inclusion criteria were as follows: (1) Studies reporting posterior shoulder dislocation; (2) Studies reporting patients with a reverse Hill-Sachs (or McLaughlin) lesion and eventual posterior glenoid margin lesions; (3) Patients who were treated with a humeral-sided surgical procedure with possible accessory procedures on the glenoid; (4) Articles reporting clinical outcomes; (5) Minimum follow-up of 9 months. 

The exclusion criteria applied were: (1) Studies reporting multidirectional instabilities of the shoulder; (2) Studies reporting associated fractures (excluded Mc Laughlin lesions); (3) Studies with less than 5 patients included and case reports; (4) Studies older than 20 years; (5) Studies with a minimum follow-up of less than 9 months; (6) Studies not published in the English language. 

### 2.4. Data Extraction

The data were extrapolated from the articles using a standardized data collection form. The data collected included: the year of publication, type of clinical study, level of evidence (I–IV), methods used for diagnosis, type of surgical treatment, study period, inclusion/exclusion criteria, number of patients enrolled, number of patients available for follow-up, age, length of follow-up, proportion of dominant extremities involved, development of osteoarthrosis (OA), and different clinical outcomes reported in each study, including post-operative range of motion and clinical scores. 

The functional outcomes scores that were included in our study were the Constant–Murley (C–M) outcome score [15]; the University of California, Los Angeles (UCLA) outcome score [16]; Rowe score; Simple Shoulder Test (SST) [17]; the Disabilities of the Arm, Shoulder, and Hand score (DASH) [18]; and visual analogue scale (VAS) for pain [19]. Finally, the presence of bias was determined and analyzed for each eligible study. 

### 2.5. Quality Assessment

Two authors (F.C. and A.F.) used the methodological index for non-randomized studies (MINORS) criteria to assess the quality and the risk of bias. The ideal global score is 16 for non-comparative studies and 24 for comparative studies. The items were scored 0 if not reported, 1 if reported but inadequate, and 2 if reported and adequate. If no consensus was reached between the evaluation of the two authors mentioned above, the independent opinion of a third reviewer was decisive (VGR). The MINORS scores are reported in Table 1. The quality of the included studies was based on the Strengthening the Reporting of Observational studies in Epidemiology (STROBE) checklist criteria, which is a reliable quality rating tool for observational studies [20]. Each criterion was scored as “yes”, “no”, or “not applicable (NA)”. A criterion was scored as “yes” if it was applicable and met in the study, “no” if it was applicable but not met, and “NA” if it was not relevant to the study. The scores obtained were compared among the reviewers to assess the importance and validity of each individual study. The number of criteria scored as “yes” divided by the number of applicable criteria per manuscript yielded a percentage of the applicable STROBE criteria. 

All the articles included in our review had a STROBE percentage score greater than 90%, as shown in Table 1. This data highlights the quality of each study even if the level of evidence of each study was not greater than III. 

### 2.6. Statistical Analysis

The results were summarized using descriptive statistics for continuous variables and frequencies and percentages for categorical variables. All data were collected in Microsoft Excel, 2016 version (Microsoft Corporation, Redmond, WA, USA). The data analysis was performed with IBM SPSS Statistics for Windows, Version 25.0 and Review manager software (RevMan, version 5.3). Continuous variables have been reported as the mean with range or standard deviation. Weighted means have been calculated for cohort studies and case reports.

## 3. Results

### 3.1. Study Population and Demographics

Our first database research produced 283 studies using Pubmed, Embase, and Google Scholar. In total, 9 studies were added through cross references and 151 papers were excluded as duplicates found in different databases: altogether, 141 studies were obtained. We then conducted a title and abstract analysis to obtain 43 papers that were screened using our inclusion and exclusion criteria. The whole inclusion/exclusion process is summarized in the PRISMA flowchart in Figure 1 [13]. Sixteen studies met the inclusion and exclusion criteria of our review. 

The final population included 207 shoulders with a weighted average age of 41.7 years. The clinical and radiological results were reported with a weighted mean follow-up of 62.1 months. The shortest follow-up period (18 months) was reported by Abdel-Hameed et al. [21], whereas Gerber et al. [22] and Konrads et al. [23] reported the longest one with a mean value of 128.4 months. Schliemann et al. [24] reported the results of 35 patients with a posterior shoulder dislocation who were treated with different approaches and methods; the demographical data reported refer to the entire population regardless of the treatment method used. 

**Table 1:** Quality assessment and Study characteristics. PCS: Prospective Case Series; RCS: Retrospective Case Series; MINORS: methodological index for non-randomized studies; STROBE: Strengthening the Reporting of Observational studies in Epidemiology, the result is reported as a percentage; Age: shown in years (range) or ± standard deviation if reported; Follow-up: shown in months (range) or ± standard deviation if reported; S. Technique: Surgical technique; MML: Modified McLaughlin procedure; ARR: Arthroscopic Reverse Remplissage; ML: McLaughlin procedure; W.A.: Weighted Average.

	Study Design	MINORS	STROBE	Shoulders	Age	Follow-Up	S. Technique
*Abdel-Hameed 2015 [21]*	PCS	8	90.9	9	29.5 (22–46)	18 (16–21)	MML
*Banerjee 2013 [25]*	RCS	10	90.9	7	39 (22–60)	41 (27–54)	MML
*Cohen 2022 [26]*	RCS	10	90.9	10	36.3 (23–54)	59.4 (24–110)	MML
*Demirel 2017 [27]*	RCS	10	90.9	13	39.3 (28–72)	30 (12–67)	MML
*Diklic 2010 [8]*	RCS	10	90.9	13	42 (36–51)	54 (41–64)	Graft
*Gavriildis 2008 [28]*	RCS	8	90.9	12	49.8 ± 8.6	37.4 ± 6.8	Arthroplasty
*Gerber 2014 [22]*	RCS	8	95.4	22	44 (25–75)	128.4 (60–294)	Graft
*Khira 2017 [29]*	PCS	10	90.9	12	26 (22–36)	30 (24–48)	MML
*Konrads 2023 [23]*	PCS	12	95.4	12	39 (20–55)	128.4 (111.6–153.6)	Cartilage elevation/Graft
*Martinez 2012 [30]*	RCS	10	90.9	6	31.7 (28–36)	122 (96–144)	Graft
*Marcheggiani Muccioli 2021 [6]*	RCS	12	90.9	12	54.8 (31–72)	66 (24–225)	Graft
*Mittal 2022 [31]*	RCS	10	90.9	16	34.6 (27–64)	27	MML/Arthroplasty
*Romano 2021 [5]*	RCS	10	95.4	12	32.8 (22–45)	37.3 (24–58)	ARR
*Schliemann 2011 [24]*	RCS	12	95.4	35	53 (30–86)	55 (11–132)	Graft/ML/Osteotomy
*Shams 2016 [32]*	PCS	12	90.9	11	39 (31–49)	29 (24–39)	MML
*Xiong 2023 [33]*	PCS	10	90.9	5	51 (27–81)	19.8 (12–30)	MML
*W.A.*				207 (total)	41.7	62.1	

In Table 2, the patient’s demographics and the surgical approach characteristics are summarized. The Modified McLaughlin (MML) procedure was the most commonly used surgical technique, with a total of 8 studies reporting the results of this surgical technique. A surgical treatment involving bone graft use was the second most commonly performed procedure to assess the McLaughlin lesion. The results of these two techniques will be analyzed individually below in two different paragraphs.

Of the 207 shoulders included in our review, the majority (107) have suffered a posterior shoulder dislocation after a traumatic onset. The second most frequent onset described was after either a seizure or electrical shock. Diklic et al. [8] reported data from one patient who fell after a seizure; therefore, the reason for the posterior shoulder dislocation could not be determined. Three studies [5,6,33] did not report the cause of the dislocation of their population. 

In our analysis, we found a large consensus about the imaging used for the diagnostic process. All of the studies included—except for two [5,8]—used both X-rays and a CT scan. Konrads et al. [23] performed a diagnostic arthroscopy as the first part of their surgical procedure in order to assess the cartilage damage: if the patient was treated within 14 days and a cartilage lesion of less than grade 2 (ICRS classification) was detected, the joint surface was restored using a retrograde elevation of the McLaughlin lesion. If worse cartilage damage was found (Grade 3 or 4 ICRS), an open surgical procedure was performed. Romano et al. [5] performed an MRI instead of a CT scan in addition to plain X-rays for diagnosis. Marcheggiani Muccioli et al. [6] performed an MRI in addition to X-rays and CT in the diagnostic process. 

The size of the reverse Hill-Sachs lesion was measured mainly on the CT scan or on the MRI. The measurement technique described by Gerber et al. [22] was used five times, whereas the method described by Moroder et al. [34] was used two times. Some studies defined the lesion dimension on X-rays without providing information on their used method [8,24]. 

### 3.2. Overall Results

Our review included 16 articles, for a total of 215 shoulders (165 males), of which 202 completed the scheduled follow-up. The clinical and radiological results are shown in Table 3. The weighted average age was 39.9 (26–54.8) years old. The average weighted time to surgery was 16.9 (7.5–58) weeks and was reported in 9 out of 16 studies. Onset was evidenced for 126 shoulders and was divided as follows: 52 seizures, 67 traumas, and 7 had other causes (4 were caused by electrocution). All studies reported the pre-operative imaging used: pre-operative X-rays were performed in all studies, 13 studies also used CT scans, while MRI was used in 2 studies only. Romano et al. and Konrads et al. also performed a diagnostic arthroscopy in the surgical setting [5,23]. Measurements of the defect were performed, adopting different techniques: one author used the Chen method, 4 used the Gerber method, 2 used the Moroder method, and 1 used the Hawkins method. The average weighted Reverse Hill-Sachs lesion dimension was 35.2 (20–50.4%). All Mclaughlin procedures, Modified Mclaughlin procedures, and graft procedures were performed on defects < 50% of the humeral head, while a joint replacement was performed in other cases.

Six articles reported a graft procedure and eleven ML e MML procedures. Gavriildis et al. [28] reported results for 12 joint replacements (average RHS lesion was >50%), Mittal et al. [31] also described 5 hemi-replacement arthroplasty (HRA), 3 reverse shoulder arthroplasty (RHS lesions > 50%), a MML along with great tuberosity osteotomy, a MML along with metaphyseal osteotomy. Romano et al. [5] described only arthroscopic ML procedures, Schliemann et al. [24] reported 6 rotational osteotomies (2 performed with MML).

The average weighted follow-up time was 55.5 months (18–128.4); calculations could not include one paper because it reported follow-up only as a minimum time [31].

Pre-operative weighted average flexion was 53.4° (20–90°). Post-operative weighted average flexion was 137.1 (107.1–157.7). Pre-operative abduction was reported only by 4 studies [28,31,32,33]; the weighted average was 44.4° (13.3–95); and the post-operative abduction weighted average was 136.6 (95.8–171.4). 

Pre-operative weighted average external rotation with the adducted arm was 1.3° (−6.7–8.3°) and post-operatively it increased to 45.4° (35–79.2). Eight studies reported the external rotation range with the abducted arm and the average post-operative value was 72.2° (51–83.6) [5,6,21,25,26,29,32,33]. Pre-operatively average weighted internal rotation was 9.4° (1–25) and was reported in three studies [5,29,31]. Post-operatively average weighted internal rotation with the adducted arm was 60.9° (45–80) and data were reported in six studies [5,21,29,30,31,32]. Other authors did not report the average values of their studies, or only reported vertebral internal rotation scores.

Several clinical scores were used: Constant Murley Score (CMS), American Shoulder and Elbow Surgeons (ASES) score, University of California, Los Angeles (UCLA) activity score; Subjective Shoulder Value (SSV) score, ROWE score, Walch score, Disability of the Arm Shoulder and Hand (DASH) score, Western Ontario Shoulder Instability Index (WOSI), Visual Analog Score (VAS). Only three studies reported the pre-operative CMS with a mean value of 29 points [5,26,33]. The weighted average CMS found at the post-operative clinical evaluation was 79.4 (59–94) and was reported in 9 studies [5,8,22,23,24,26,27,28,33]. Joint replacement procedures [28] and rotational osteotomy associated with ML procedure [24] reported the lowest post-operative scores, respectively, 59.5 and 59.

Pre-operative ASES was reported only by one study with a weighted average of 48.1 (32–34), while post-operative ASES was reported in four studies and its weighted average was 82.5 (63.2–98) [6,25,27,31]. 

Pre-operative UCLA score was reported in four studies and its weighted average was 15.7 (7.8–25.8) [21,26,31,32]. The post-operative weighted average UCLA was found to be 27.4 ± 5.2 (17–31) and was reported in five studies [21,26,29,31,32]. 

The results of the remaining clinical scores used are reported in Table 4. 

Pre-operatively, VAS was reported in two studies showing an average weighted score of 3.4 (2.4–4.6) [26,31]. The post-operatively average weighted VAS score was 2.0 (1.0–2.4) [23,26,31]. Diklic et al. and Gerber et al. reported the CMS pain score to be, respectively, 12.5 and 14 out of 15 [8,22].

Pre-operative osteoarthrosis was only observed by Romano et al.’s arthroscopic evaluation. According to Samilson–Prieto he observed 2 grade I and 1 grade II [5]. Post-operatively, Romano et al. reported 4 grade I, 1 grade II, while Cohen et al. reported 6 grade I, 2 grade II, 2 grade III, and Marcheggiani Muccioli et al. reported 10 grade I and 2 grade II at final follow up [5,6,26]. Demirel et al. used Kelgren and Lawrence classification and reported 0 post-operative osteoarthrosis [27]. Gerber et al. described 4 mild cases and 4 advanced cases of developed osteoarthrosis at final follow-up [22].

Complications were reported as follows: Banerjee et al. reported 1 screw migration in a patient whose internal rotation was limited; Diklic et al. observed 1 osteonecrosis at follow-up; Gerber et al. reported a shoulder replacement dissociation (polyethylene-inlay) requiring revision 36 weeks later and 3 persistent posterior shoulder subluxations (2 mild, 1 severe); Martinez et al. reported 2 head collapses with avascular necrosis and severe osteoarthritis in a patient 8 years post-operatively, requiring reverse shoulder replacement; Xiong et al. observed one patient reporting a constant severe pain [8,22,25,30,33].

### 3.3. McLaughlin and Modified McLaughlin Procedure Results

Ten studies were included, reporting the results for 93 shoulders (59 males) that underwent the Modified Mclaughlin surgical procedure or Mclaughlin surgical procedure for posterior instability: a total of 74 Modified Mclaughlin and 19 Mclaughlin procedures were analyzed in our review. The weighted average age of the population examined was 33.6 (29.5–53) years old (only 6 studies reported specific data for the age of the patients treated). The average weighted time to surgery was 14.9 (9–56) weeks. Schliemann et al., Shams et al., and Romano et al. [5,24,32] did not report dislocation onset, while other authors reported 34 dislocations due to seizures, 57 to traumas, 6 for other reasons (of which 3 due to electrocution).

Eight studies reported the imaging investigations patients underwent before surgery: all studies used plain Radiographs and CT scans, but methods of measurement of the defect were different. In total, 3 authors used the Gerber method, 1 used the Moroder method, 1 used the Chen method, 1 used the Cicak method, and the other authors did not specify their method. The weighted average showed, despite calculation methods of the defect being different, reverse Hill Sachs to be 31.2%; lesions were always less than 50%, while the minimum was 22%. Modified Mclaughlins were performed using some variations: Banerjee et al. reported transferring either the whole lesser tuberosity and subscapularis tendon into the defect or its upper two-thirds, based on the measured defect entity. Mittal et al. reported, in one case, a malunion of the greater tuberosity and in another case a malunion of the metaphysis, therefore, they performed an osteotomy in the first case of the greater tuberosity without detaching the cuff, repositioning the segment in its original position by using tension band wiring while a metaphyseal osteotomy was performed in the second case to correctly orientate the greater tuberosity, finally applying a locking plate. At the end of these two procedures, a Modified McLaughlin was performed [25,31]. Schliemann et al. described a rotational osteotomy as well as a Modified McLaughlin procedure on 2 shoulders [24]. Romano et al. described an arthroscopic reverse remplissage of the subscapularis [5].

The fixation of the bone segment was achieved with different instruments and techniques: two studies reported fixing the tuberosities with two 4 mm cannulated screws [27,29]; one used 1 or 2 bicortical partially threaded cannulated self-tapering 3.5 mm screws [25]; one used a double row technique (anchors along with transosseous sutures) [26]; two studies used 2–3 transosseous horizontal sutures in ethibond 5-0 [21,32]; one used two non-specified screws for the lesser tuberosity [31]. Romano et al. described using different fixation methods: if the defect was less than 20% a 5 mm triple loaded suture was used, if the defect was more than 20% a double anchor, it was fixed with a sliding locking knot through subscapularis with 3 half stitches [5].

Accessory procedures regarded biceps long head tenodesis, and the detachment of great pectoral muscle and osteotomies. Three studies reported continually performing long biceps head tenodesis (30 shoulders), one author always detached the great pectoral muscle and performed a posterior capsular release (9 shoulders) [21,26,27,31]. Mittal et al. also reported one shoulder with great tuberosity and metaphyseal malunion and performed a corrective osteotomy as previously mentioned [31]. Romano 2021 described posterior capsulolabral repair [5].

Post-operative indications reported a neutral rotation for 6 weeks for 20 shoulders, 30° abduction for 6 weeks for 13 shoulders, 45° extra rotation for 4 weeks for 7 shoulders, and 23 shoulders were immobilized as well in external rotation for 4 weeks without the author expressing the exact degree of rotation the arm was kept on.

Average weighted follow up of the studies was 33, 1 month (18–59.4). Schliemann et al. was excluded in our calculations because data were not divided in subgroups [24].

Pre-operative range of motion showed the average weighted flexion (61 shoulders) to be 74.1° (70–90); abduction (16 shoulders) to be 80.9° (50–90), external rotation (27 shoulders) to be 3° (3–7). Internal rotation was reported both with adducted and abducted arm and 15 shoulders could reach at the gluteus, 20 at L5, while Khira et al. reported a pre-operative average internal rotation of 25° and Romano et al. a 1° internal rotation in their patients [5,21,26,29,32,33]. Post-operative average weighted range of motion was: flexion (79 shoulders) 159.7° (126–175,7), abduction (57 shoulders) 149.6° (130–171.4), external rotation (79 shoulders) 70.6° (51–83.6), internal rotation was measured to be 56.9° in 44 shoulders (45–80) or in the vertebral system: L1 for 2 shoulders, T7 in 4 shoulders, T12 for 4 shoulders, L2 for 5 shoulders, L3 for 3 shoulders, 2 at the gluteus, 7 between gluteus, and T12.

Clinical shoulder scores were reported as follows: average weighted pre-operative CMS (27 shoulders, [5,26,33]) 29.1 (22–46); post-operative (47 shoulders) 75.8 (65–94); only Mittal et al. reported pre-operative ASES at 33.2 (30–36), while the post-operative average weighted ASES (27 shoulders) was 84.4 (78–98) [31]; the pre-operative average weighted UCLA (28 shoulders) was 13 (7.9–25.8), the post-operative UCLA on 49 shoulders (reported by Abdel Hameed et al., Cohen et al., Khira et al., Mittal et al., Shams et al.) was 37 (26.8–31) [21,26,29,31,32]. Schliemann et al. evaluated post-operative ROWE to be 75 on 5 shoulders which underwent the Mclaughlin procedure and 60 on 2 shoulders which underwent the Modified Mclaughlin procedure. Cohen et al. reported pre-operative VAS to be 4.6 on 10 shoulders, Mittal et al. reported it to be 2.6 on 7 shoulders, and, respectively, it was reported to be post-operatively 2.4 and 1 [24,26,31]. Pre- and post-operatively SSV was only reported by Romano et al. and it was, respectively, found to be 30% and 93%. Romano et al. was the only study to report the WOSI score pre- and post-operatively: 41 and 92, respectively [5].

Post-operative osteoarthritis was reported by Cohen et al. to be during the follow-up grade 1 on 6 shoulders, grade 2 on 2 shoulders, and grade 3 on 2 shoulders, according to the Samilson–Prieto Score System [26]. Romano et al. reported post-operatively 4 grade 1 and 1 grade II (according to Samilson–Prieto); they also reported MRI 1 chondral degeneration and 3 increase of glenohumeral osteoarthritis [5]. Schliemann et al. reported narrowing of the joint space and the presence of previously undetected osteophytes in 68% of shoulders. They also noted decreased acromiohumeral distance in 34% of shoulders [24]. Banerjee et al., Demirel et al., Shams et al., and Xiong et al. reported no post-operative osteoarthritis [25,27,32,33].

Banerjee et al. reported 1 screw migration which limited external rotation, Abdel Hameed et al. reported 1 fair result and 1 poor result, Shams et al. reported 1 patient who sustained a constant pain [21,25,32]. Romano et al. reported osteoarthritis incidence both pre- and post-operatively. Pre-operatively, he reported 2 grade I and 1 grade II [5]. Cohen et al. reported 0 revision surgeries, although the 2 shoulders were needed an arthroplasty years later due to a previous posterior dislocation late diagnosis [26]. 

### 3.4. Graft Surgical Procedures Results

In Table 5, we have resumed the results of the surgical graft procedures. Six papers reported the clinical and radiological results of surgical techniques that involved a bone grafting procedure with a total of 71 shoulders treated with this technique [6,8,22,23,24,30]. The weighted average follow-up was 92.3 months. The average CM score was found to be 83.7 points. 

A Femoral Head allograft was the most commonly used, with a total of 27 patients treated with it. Three studies did not report the type of allograft used [23,24,30]. 

The lesion dimension was measured 3 times using the Gerber method and one paper used the method described by Moroder et al. [22,34]. Schlieman et al. reported the population treated with an allograft with the smallest reverse Hill-Sachs lesions ranging between 15 and 25% of the humeral head. 

Konrads et al. did not report any imaging method used at follow-up for the radiological evaluation of the outcome [23]. The other 5 authors used X-rays and Martinez et al. and Marcheggiani Muccioli et al. also performed a CT scan at the final follow-up evaluation [6,30].

Post-operative osteoarthrosis was developed by many patients as shown in Table 5. Gerber et al. observed 7 cases of new osteoarthritis after an allograft transplantation surgery, 5 of which were treated in a chronic setting [22]. Marcheggiani Muccioli et al. described post-operative mild osteoarthritis (Grade I-II of Samilson–Prieto classification) in 12 patients out of 12, with 2 Grade II and 10 Grade I osteoarthritis described [6].

Revision surgery was performed in 3 patients in Gerber et al.’s population and in 2 patients in Martinez et al.’s [22,30]. 

## 4. Discussion

The most important findings of this systematic review highlight the complexity and diversity of treatment strategies, underscoring the necessity of a tailored approach based on individual patient factors and lesion characteristics.

In particular, in patients affected by posterior shoulder dislocation, the Modified McLaughlin procedure is the most commonly employed surgical technique, followed by the use of bone grafts. Within the sixteen articles incorporated into our study, eight employed the McLaughlin technique [21,25,26,27,29,32,33] and five utilized bone grafting [6,8,22,24,30]. 

On the other hand, the innovative approach of employing diagnostic arthroscopies, as recently advocated by Konrads et al. [23], is gaining notable acceptance within the orthopedic community. This method stands out due to its minimally invasive nature, which results in reduced patient morbidity and quicker recovery times compared to traditional open surgical methods.

This diversity in surgical methods reflects the tailored approach to treating the specific pathology presented in each case. The McLaughlin technique, often chosen for its reliability and proven outcomes, was the predominant method. Bone grafting was also a common choice, particularly in scenarios requiring structural support due to bone loss. The less frequently used techniques, including arthroscopic cartilage elevation and prosthetic replacement, represent the evolving landscape of surgical interventions, offering alternatives when traditional methods are contraindicated or have failed. This variation in techniques underscores the importance of a personalized treatment strategy in orthopedic surgery, guided by the nature and severity of the joint condition as well as patient-specific factors.

However, our study highlights a preference in the surgical field for head-preserving approaches and graft-based repair techniques, rather than prosthetic replacement in managing McLaughlin lesions.

The incidence of posterior shoulder dislocations caused by trauma was the highest in our study.

This significant finding highlights the need for healthcare professionals and policymakers to focus on reducing the incidence of trauma through public health interventions and improved safety protocols.

The second most common cause was seizures or electrical shocks, suggesting that these conditions are significant risk factors for posterior shoulder dislocation.

This suggests that individuals with conditions predisposing them to seizures or those who are at risk of electrical shocks may require special attention in terms of preventive strategies and emergency care protocols.

Therefore, 3 studies [5,6,33] did not report the cause of the dislocation of their population.

This omission highlights a critical area for future research to address, ensuring a more comprehensive understanding of the causes of shoulder dislocations.

Another notable aspect emerging from this study is the extensive and consistent use of diagnostic imaging, particularly the combination of X-rays and CT scans. Advanced imaging techniques like MRI and CT scans have been instrumental in pre-operative planning, allowing for a more accurate assessment of the lesion’s size and the extent of associated soft tissue injuries. In particular, the use of MRI by Romano et al. [5] and Marcheggiani Muccioli et al. [6] reflects a trend towards more advanced diagnostic methods, which may offer better assessment of cartilage injuries and soft tissue.

The size of the reverse Hill-Sachs lesion was measured mainly on CT scans or MRIs, with variations in measurement techniques among studies.

Notably, time to surgery post-dislocation varied significantly across the studies, ranging from less than three days [23] to over 160 days [28], illustrating the challenges in standardizing the timing of intervention. Surgical procedures varied, with options such as the McLaughlin procedure, Modified McLaughlin procedure, graft procedures, joint replacements, and osteotomies being performed based on the extent of the lesions.

The size of the reverse Hill-Sachs lesion played a pivotal role in determining the choice of surgical technique. For lesions smaller than 50% of the humeral head, Modified McLaughlin procedures were favored. In contrast, graft procedures and joint replacements were considered for larger defects. Notably, graft procedures using allografts, particularly femoral head allografts, were utilized in several studies. These procedures demonstrated favorable clinical outcomes, with average CMS scores indicating substantial post-operative improvement. However, it is essential to acknowledge that some patients developed post-operative osteoarthritis, highlighting the importance of long-term follow-up and ongoing evaluations of joint health.

Moreover, our review revealed that clinical scores, including the Constant Murley Score (CMS), American Shoulder and Elbow Surgeons (ASES) score, and University of California, Los Angeles (UCLA) activity score, showed substantial improvements post-operatively. These improvements indicate the effectiveness of surgical interventions in restoring shoulder function and reducing pain overall. However, it is important to note that joint replacement procedures and rotational osteotomies were associated with lower post-operative scores, suggesting that these techniques may have limitations in achieving optimal functional outcomes.

Complications were reported in some cases, including screw migrations, osteonecrosis, and shoulder subluxations. These findings underscore the need for careful surgical planning and meticulous execution to minimize potential complications. The development of osteoarthritis post-operatively was noted in some cases, with varying degrees of severity.

## 5. Limitations

The heterogeneity in surgical approaches and methodologies identified in our systematic review highlights an inherent limitation within our study. While this diversity reflects the real-world spectrum of clinical practice and individualized patient care, it also presents challenges in synthesizing data to derive conclusive outcomes. The absence of standardized protocols across the studies included in our analysis precludes a direct comparison of results and may contribute to variability in patient outcomes.

Moreover, the discrepancies in surgical techniques not only confound the evaluation of efficacy but also complicate the determination of the best practice guidelines. The lack of uniformity in outcome measures further exacerbates the difficulty in assessing the true comparative effectiveness of each technique. Such variation might affect the generalizability of our findings and underscore the necessity for consensus on surgical management and standardized reporting in future research.

## 6. Conclusions

In conclusion, our study summarizes the different surgical possibilities to treat the reverse Hill-Sachs lesion based on the lesion characteristics themselves. The Modified Mc-Laughlin procedure is still one of the most utilized and successful procedures, but the use of the Allograft treatment is a valuable option that leads to similar clinical and radiological results. Future research could focus on standardizing surgical procedures and developing evidence-based guidelines to optimize outcomes for patients with posterior shoulder dislocation. Further studies should also explore the long-term impact of different surgical techniques on shoulder functionality and patient quality of life.

## Figures and Tables

**Table 2:** Demographics and Surgical Technique. T: Trauma; S: Seizure or electric shock; T + S: Trauma and Seizure combined; N.D.: Undefined onset; Imaging: Imaging used for Diagnosis; CT: Computed Tomography; MRI: Magnetic Resonance Imaging; Lesion measure: Data shown as percentage ± standard deviation (range) (Measurement technique) if available. Time to Surgery: time between the shoulder dislocation and the surgical treatment. Time is reported in weeks (range) if not described differently. W: weeks; *: Only the time between the shoulder dislocation and the diagnosis was reported; S. Technique: Surgical technique; MML: Modified McLaughlin procedure; ARR: Arthroscopic Reverse Remplissage; ML: McLaughlin procedure.

	Shoulders	Onset	Imaging	Lesion Measure (%) (Technique)	Time to Surgery	S. Technique
*Abdel-Hameed 2015 [21]*	9	4 T., 3 S., 2 N.D.	X-ray + CT	40 (35–45)		MML
*Banerjee 2013 [25]*	7	6 T., 1 S.	X-ray + CT	32 ± 6.4 (25–45)	<14 days	MML
*Cohen 2022 [26]*	10	5 T., 5 S.	X-ray + CT	32 (22–35)	23 (6–61)	MML
*Demirel 2017 [27]*	13	4 T., 9 S.	X-ray + CT	27 (20–40)		MML
*Diklic 2010 [8]*	13	3 T., 9 S., 1 T + S	X-ray	25–50 (Measured on X-ray)	17.14 (8.6–38.6)	Graft
*Gavriildis 2008 [28]*	12	7 T., 5 S.	X-ray + CT	>45	160.3 ± 72	Arthroplasty
*Gerber 2014 [22]*	22	12 T., 10 S.	X-ray + CT	43 (30–55) (Gerber)	9 < 30 days + 13 27w (4.3–64.3)	Graft
*Khira 2017 [29]*	12	9 T., 3 S.	X-ray + CT	40 (30–45)	8 (4–13)	MML
*Konrads 2023 [23]*	12	11 T., 1 S.	X-ray + CT + Arthroscopy	25–40 (Moroder)	9 < 14 days; 3 > 14 days	Cartilage elevation/Graft
*Martinez 2012 [30]*	6	3 T., 3 S.	X-ray + CT	>40 (Gerber)	7.5 (7–8)	Graft
*Marcheggiani Muccioli 2021 [6]*	12		X-ray + CT + MRI	30–50 (Gerber)	<3	Graft
*Mittal 2022 [31]*	16	14 T., 2 S.	X-ray + CT	<50 or >50	8.6–30	MML/Arthroplasty
*Romano 2021 [5]*	12		X-ray + MRI	20 ± 9.6	14.3 (4–48)	ARR
*Schliemann 2011 [24]*	35	26 T., 9 S.	X-ray + CT	0–25/>25 (Measured on X-ray or CT)	9.43 (0–52.1) *	Graft/ML/Osteotomy
*Shams 2016 [32]*	11	3 T., 7 S., 1 N.D.	X-ray + CT	35 (30–40)	9 (3–18)	MML
*Xiong 2023 [33]*	5		X-ray + CT	36.2 (30–40)	11.6 (6–24)	MML
	207	107 T. 67 S. 1 T. + S. 3 N.D.				

**Table 3:** Clinical and Radiological results. T.t.S.: time to surgery—time between the shoulder dislocation and the surgical treatment. Time is reported in weeks, if not described differently. d: Days *: data not included in calculations. Lesion measure: Data are shown as an average value or range and are expressed as a percentage. MML: Modified Mclauglin technique; J. Rep: Joint Replacement procedure; HRA: Hemi-replacement Arthroplasty; RSA: Reverse Shoulder Arthroplasty; GTO: Great Tuberosity Osteotomy; MO: Metaphyseal Osteotomy; RO: Rotational Osteotomy—specific data for the treatment group were not available; CMS: Constant Murley Score; ASES: American Shoulder and Elbow Score; UCLA: University of California, Los Angeles; VAS: Visual Analog Scale; DASH: Disability of the Arm, Shoulder, and Hand; SSV: Subjective Shoulder Value; Not available (NA): Data are not reported.

	Patients	T.t.S.	Lesion Measure	S. Technique	CMS	ASES	UCLA	VAS	Other Score
*Abdel-Hameed 2015 [21]*	9	NA	40.0	MML			31.0		
*Banerjee 2013 [25]*	7	14.0	39.5	MML		98.0			
*Cohen 2022 [26]*	10	23.0	32.0	MML	65.0		27.0	2.4	
*Demirel 2017 [27]*	13	NA	27.0	MML	85.0	78.0			
*Diklic 2010 [8]*	13	17.1	37.5	GRAFT	86.8			12.5	
*Gavriildis 2008 [28]*	12	58.0	50.5	J. REP	59.5				
*Gerber 2014 [22]*	19	9 < 30 d. + 13 27w (4.3–64.3) *	43.0	GRAFT	77.0			14	SSV: 88%
*Khira 2017 [29]*	12	8.0	40.0	MML			30.0		
*Konrads 2023 [23]*	10	9 < 14 d; 3 > 14 d *	25–40	GRAFT	92.5			1.9	DASH: 3.2 SSV: 91
*Martinez 2012 [30]*	6	7.5	>40 *	GRAFT					
*Marcheggiani Muccioli 2021 [6]*	12	<3 *	30–50	GRAFT		94.0			
*Mittal 2022 [31]*	5	8–30 *	<50 or >50 *	MML		82.9	26.8		
	5	-	-	HRA		63.2	18.8	2.0	
	1	-	-	MML + GTO		82.9	26.8	1.0	
	3	-	-	RSA		58.0	17.0	1.0	
	1	-	-	MML + MO		82.9	26.8	1.0	
	1	-	-	HRA		63.2	18.8	2.0	
*Romano 2021 [5]*	12	14.3	20.0	ML	94.0				SSV: 93%
*Schliemann 2011 [24]*	35	9.43	20.0		89.0				ROWE: 79
	11	-	-	GRAFT	89.0				ROWE: 79
	5	-	-	ML	62.0				ROWE: 75
	4	-	-	R.O	59.0				ROWE: 55
	2	-	-	R.O. + ML	70.0				ROWE: 60
*Shams 2016 [32]*	11	9.0	35.0	MML			30.0		
*Xiong 2023 [33]*	5	11.6	36.2	MML	46.0				
	Total = 202	16.9 (7.5–58)	35.2 ± 9.1		79.4 ± 13.3	82.5 ± 12.6	27.4 ± 5.2	1.9 ± 0.6	

**Table 4:** McLaughlin treatment results. T.t.S.: Time to Surgery—time between the shoulder dislocation and the surgical treatment. Time is reported in weeks. if not described differently; *: data not included in calculations. Lesion measure: Data shown as average value or range and are expressed as percentage (Measurement technique) MML: Modified Mclauglin technique; GTO: Great Tuberosity Osteotomy; MO: Metaphyseal Osteotomy; RO: Rotational Osteotomy—specific data for the treatment group were not available; CMS: Constant Murley Score; ASES: American Shoulder and Elbow Score; UCLA: University of California, Los Angeles; VAS: Visual Analog Scale; DASH: Disability of the Arm, Shoulder, and Hand; SSV: Subjective Shoulder Value; Not available (NA): Data are not reported; Osteoarthrosis: SP—Samilson–Prieto scoring system; KL: Kellgren and Lawrence #: Reduction of the acromion-humeral distance.

	Patients	T.t.S.	Lesion Measure	Surgical Technique	Clinical Outcome	Imaging	OA	Revision Surg.
*Abdel-Hameed 2015 [21]*	9	NA	40% (35–45%) (NA)	MML	UCLA 31 (27–34)	X-ray	NA	0
*Banerjee 2013 [25]*	7	<14 *	32% (25–45%) (Cicak)	MML	ASES 98 CS 92 (80–98)	X-ray	0 (SP)	1 screw removal
*Cohen 2022 [26]*	10	23 (6–61)	32% (22–35%) (Gerber)	MML	CM 65 ± 21.5	X-ray + CT	6 grade I; 2 grade II; 2 grade III (SP)	
*Demirel 2017 [27]*	13	NA	27% (20–40%) (Moroder)	MML	CM 85 ASES 78	X-ray	0 (KL)	0
*Khira 2017 [29]*	12	8 (4–13)	40% (30–45%) (% of total)	MML	UCLA 30 (28–33)	X-ray + CT	NA	NA
*Mittal 2022 [31]*	7	2–7 months	<50% (Axial plane measurement) *	5 MML; 1MML + GTO; 1 MML + MO	ASES 82.85 (78–86); UCLA 26.8 (24–30)	X-ray + CT	NA	NA
*Romano 2021 [5]*	12	14.3 (4–48)	20% ± 9.6% (“Ellipsoid drawn”)	Arthroscopic ML	CMS: 94 ± 3 SSV 93 WOSI 92 ± 4	X-ray + MRI	4 grade I, 1 grade II (SP)	0
*Schliemann 2011 [24]*	5	9.43 (0–52.1)	>25% (NA)	ML	CMS 62, ROWE 75	X-ray	68% Osteophytes, 34% decreased A-H distance #	NA
	2	9.43 (0–52.1)	>25% (NA)	ML + RO	CMS 70, ROWE 60	X-ray	/	/
*Shams 2016 [32]*	11	9 (3–18)	35% (30–40%) (Chen)	MML	UCLA 30 (20–34)	X-ray + CT	0	0
*Xiong 2023 [33]*	5	11.6 (6–2)	36.2% (30–40%) (Gerber)	MML	CMS 85.8 ± 4.9	X-ray + CT	0	0
	93	14.9 (9–6)	31.2% (22–50%)		CMS 79.4 ± 13.3 ASES 82.5 ± 12.6 UCLA27.4 ± 5.2			

**Table 5:** Graft treatment results. Lesion measure: Data are shown as an average value or range and are expressed as percentage (Measurement technique). T.t.S.: Time to Surgery—time between the shoulder dislocation and the surgical treatment. Time is reported in weeks (range) if not described differently; * Only the time between the shoulder dislocation and the diagnosis was reported; F.H.: Femural Head; I.C.: Iliac Crest; H.H.: Humeral Head; Follow-up: time shown in months; #: data referred to the entire population of the study—specific data for the Graft treatment group were not available; CMS: Constant Murley Score; Imaging: Imaging used for Diagnosis; CT: Computed Tomography; Not available: Data are not reported; OA: development of osteoarthritis; Sam.Prieto: Samilson–Prieto classification of OA.; A-H: Acromio-Humeral distance.

	Shoulders	Lesion Measure	T.t.S.	Type of Allograft	Follow-Up	CMS	Imaging	OA	Revision Surgeries
*Diklic 2010 [8]*	13	25–50 (Measured on X-ray)	17.14 (8.6–38.6)	12 Fresh frozen F.H. + 1 cryopreserved F.H.	54 (41–64)	86.8 (43–98)	X-ray	None	None
*Gerber 2014 [22]*	14	45.71 (Gerber)	9 < 30 d + 13 27w (4–60)	Fresh Frozen F.H.	143.3	77.5 (52–98)	X-ray	7	3 (out of 22) arthroplasty
*Gerber 2014 [22]*	5	35 (Gerber)	9 < 30 d + 13 27w (4–60)	I.C. autograft	87	85	X-ray	None	3 (out of 22) arthroplasty
*Konrads 2023 [23]*	10	25–40 (Moroder)	9 < 14 days; 3 > 14 days	4 elevation cartilage e cancellous bone graft + 3 autograft	128.4 (111.6–153.6)	92.5 ± 8.9	Not available	Not available	None
*Martinez 2012 [30]*	6	>40 (Gerber)	7.5 (7–8)	Frozen allogenic	122 (96–144)	69.2 (40–100)	X-ray + CT	2 graft collapse	2 Arthroplasty
*Marcheggiani Muccioli 2021 [6]*	12	30–50 (Gerber)	<3	Fresh frozen H.H.	66 (24–225)	82 (40–97)	X-ray + CT	Sam.Prieto: 2 grade II, 10 grade I; 4% Allograft resorption	None
*Schliemann 2011 [24]*	11	15–25 (Measured on X-ray or CT)	9.43 (0–52.1) *	Allograft	55 (11–132) #	89	X-ray	68% Osteophytes, 34% decreased A-H distance #	
	71				92.3	83.7			

## Data Availability

All the data reported are available to the public in the articles cited in this review.

## Supplementary Materials

The following supporting information about the methodological process of our systematic review can be downloaded at: https://www.mdpi.com/article/10.3390/jcm13072085/s1, Prisma Checklist.
